# Gene Variant of the Bradykinin B2 Receptor Influences Pulmonary Arterial Pressures in Heart Failure Patients

**Published:** 2009-02-17

**Authors:** Thomas P. Olson, Robert P. Frantz, Stephen T Turner, Kent R. Bailey, Christina M. Wood, Bruce D. Johnson

**Affiliations:** 1 Departments of Internal Medicine; 2 Biostatistics, Mayo Clinic, Rochester, MN, 55905.

**Keywords:** genetics, hemodynamics, pulmonary hypertension, heart failure

## Abstract

**Background:**

Pulmonary arterial pressure (PAP) varies considerably in heart failure (HF) despite similar degrees of left ventricular (LV) dysfunction. Bradykinin alters vascular tone and common variations in the kinin B2 receptor (BDKRB2) gene exists. We hypothesized that genetic variation in this receptor would influence PAP in HF.

**Methods:**

131 HF patients (>1yr history systolic HF), without COPD, not currently smoking, BMI < 40, without atrial fibrillation completed the study which included a blood draw for genotyping and neurohormones (ACE, A-II, Bradykinin, ANP, BNP, and catecholamines), an echocardiogram for cardiac function and systolic PAP (PAPsys).

**Results:**

Mean LVEF was 29% ± 12%, NYHA class 2 ± 1, age 56 ± 12 yr, BMI 28 ± 5 kg/m^2^. Forty-six patients (35%) were homozygous for the +9 allele, 58 (44%) were heterozygous (+9/−9) and 27 (21%) were homozygous for the −9 allele of the BDKRB2. PAPsys averaged 42 ± 13, 38 ± 12, and 35 ± 11 mmHg for +9/+9, +9/−9 and −9/−9, respectively (p = 0.03). There was a trend towards gene effect for plasma ACE with the highest values in +9/+9 and lowest in −9/−9 patients (9.5 ± 10.7, 7.1 ± 8.7, and 5.4 ± 6.4 U/L, respectively, p = 0.06). There were no differences in plasma bradykinin or A-II, LVEF, or NYHA across genotypes.

**Conclusion:**

These data suggest the +9/+9 polymorphism of the BDKRB2 receptor influences pulmonary vascular tone in stable HF.

## Introduction

Patients with heart failure (HF) often develop pulmonary venous hypertension (PH) with an associated reactive component causing elevations in pulmonary vascular resistance (PVR). Pulmonary hypertension, secondary to HF, is a common result of systolic or diastolic dysfunction which results in a hemodynamic shift to the pulmonary circulation, elevated pulmonary venous pressure, and subsequent elevation of pulmonary artery pressure (PAP) and right ventricular pressure.^1^,^2^ In addition to this classical mechanism of elevated PAP in HF, there also appears to be a reactive component linked to a number of mitogenic and vasoactive mediators. The imbalance of mitogenic (endothelin-1, interleukin-1, vascular endothelial growth factor, etc.) and vasoactive (endothelin-1, thromboxane A, serotonin, etc.) mediators result in a predominantly vasoconstrictive atmosphere and can result in structural remodeling of the vascular endothelium and underlying smooth muscle.^3^ Interestingly however, the degree of PH in HF is highly variable for a given degree of left ventricular dysfunction and disease severity, suggesting the possibility that genetic variation may influence the susceptibility to PH in HF.

As part of the renin-angiotensin-aldosterone system, bradykinin (BK) plays an important role in the cardiovascular system by influencing blood pressure and cell proliferation.^4^ Bradykinin is a potent endogenous vasodilator nonapeptide (formed of nine amino acid residues), released from plasma globulins called kininogens. In humans, the biological action of bradykinin is mediated through the activation of two principle G-protein-coupled kinin receptor subtypes, B1 and B2.^5^ The vascular B1 receptor is normally expressed very weakly but is markedly upregulated in the presence of inflammation, cardiovascular disease, and angiotensin converting enzyme (ACE) inhibition.^6^–^8^ The endothelial cell associated bradykinin B2 receptor subtype (BDKRB2) is constitutively expressed in most tissues and is considered a much stronger mediator of vasodilation through increased production and release of nitric oxide at the endothelial level, however, it is most highly expressed in the pulmonary vasculature.^9^–^11^ In humans the BDKRB2 gene has been mapped to chromosome 14q32. The gene is more than 25 kb in size and consists of three exons. The presence of a 9 bp deletion (−9) in the gene encoding the BDKRB2 is associated with expression of higher concentrations of receptor mRNA, suggesting its strong functional relevance.^12^

Despite the growing understanding that bradykinin demonstrates strong vasodilatory properties in the systemic circulation, little is known regarding the influence of bradykinin, bradykinin receptor regulation, or bradykinin receptor genotype variants on the regulation of pulmonary vascular tonality.^13^–^15^ Due to the relationships between bradykinin and systemic pressure regulation and the relative paucity of information on the genetic interactions in the pulmonary circulation, the purpose of this study was to test the hypothesis that HF patients homozygous for the +9 polymorphism of the BDKRB2 would exhibit elevated levels of systolic PAP.

## Methods

### Population characteristics

Patients were recruited prospectively from the Mayo Clinic heart failure service and the Cardiovascular Health Clinic over the period of 2000 to 2004. Inclusion criteria included all of the following: patients with a history of ischemic or dilated cardiomyopathy, stable HF symptoms (>3 months), duration of HF symptoms >1 year, left ventricular ejection fraction (EF) ≤50%, body mass index (BMI) <40 kg/m^2^, and non-smokers with a smoking history <15 pack-years. Patients were treated with standard optimized medications for HF at the time of the study. All participants gave written informed consent after being provided a description of study requirements. The study protocol was approved by the Mayo Clinic Institutional Review Board; all procedures of this investigation conformed to the principles outlined in the Declaration of Helsinki and followed Institutional and Health Insurance Portability and Accountability Act guidelines.

### Echocardiographic evaluation

Resting supine Doppler and 2D echocardiographic measurements were performed at rest in the supine position according to the recommendations of the American Society of Echocardiography.^16^ Images were acquired with an echocardiographic ultrasound system (Vivid 7, General Electric) and stored for off-line analysis (EchoPac software, version 4.0, General Electric). Maximal early velocity (E), maximal late velocity (A), early to late velocity ratio (E/A), and deceleration time of the transmitral inflow during diastole were obtained from a 2-dimensional apical window. We measured PAP-sys as the sum of the highest velocity derived pressure gradient of the tricuspid regurgitant jet recorded from either the apical or parasternal positions and the estimated pressure of the right atrium evaluated from the inferior vena cava diameter and collapse using the simplified Bernoulli equation.^17^–^20^

Left atrial (LA) dimension, left ventricular (LV) mass, LV internal dimension during systole and diastole (LVIDs and LVIDd, respectively), LV posterior wall thickness and left atrial end-diastolic dimension were assessed by 2D apical 2- and 4-chamber views. Estimates of mass were calculated using the formula of Troy and colleagues.^21^ Left ventricular mass index was calculated as the quotient of LV mass and body surface area. Stroke volume (SV), cardiac output (CO), and LV mass were calculated according to the methods of Lewis et al.^22^ The modified Simpson’s rule was used to calculate EF.^23^

### Measurement of plasma neurohormones

Blood was drawn into Vacutainer tubes with and without EDTA and chilled until centrifuged at 4 °C at 2500 rpm for 10 minutes. Serum and plasma aliquots (1 mL) were transferred into 12 × 75 mm polystyrene tubes and frozen at −80 °C until assayed. Plasma angiotensin converting enzyme (ACE) activity was determined by spectrophotometric methods using reagents from Sigma (St. Louis, MO). Dopamine, epinephrine, norepinephrine, angiotensin II (A-II), and atrial natriuretic peptide (ANP) were assessed according to standard methods developed in the Mayo Clinic immunochemical core laboratory. Plasma bradykinin was measured by radioimmunoassay (Phoenix Pharmaceuticals, Belmont, CA). Brain natriuretic peptide was measured by specific immunoradiometric assay using two monoclonal antibodies against human BNP, one recognizing a carboxy-terminal sequence and the other the ring structure of human BNP.

### BDKRB2 genotyping

Buffy coat was obtained from whole blood collected in EDTA treated tubes and extracted using the Gentra Systems Puregene^®^ DNA Isolation kit. Polymerase chain reaction (PCR) was enhanced for high G/C content with Q solution (Qiagen, Valencia, CA) and conducted using the following primer sequence; (forward) 5′-GCC CTT GAA AGA TGA GCT G-3′ and (reverse) 5′-AAC TCC CCA CGA CCA CAG-3′.^24^ Briefly, 6.25 microliters Qiagen HotStartTaq 2X Master Mix in a final volume of 12.5 microliters including 15 ng of DNA, 1.5 mM magnesium chloride, 1.25 U HotStarTaq DNA polymerase, 200 μM each dNTP, and 5 pmoles of each primer was combined. After an initial 15 minutes at 95 °C to activate the HotStarTaq, the DNA was amplified by 35 cycles of 30 seconds at 94 °C, 30 seconds at 60 °C, and 1 minute at 72 °C. These cycles were followed by 10 minutes at 72 °C for final extension of PCR products. The PCR products were purified by ultrafiltration and sequenced in the Mayo Molecular Core Facility by a dye terminator sequencing reaction. The −9 allele produced a 266 bp PCR product whereas the +9 allele produced a 275 bp product.

### Statistical analysis

Statistical analysis and graphic presentation were accomplished using SAS (v8.2, SAS Institute, Cary, NC) to compare differences across genotypes. Univariate linear regression models and Mantel Haenszel chi-squared tests for linear trends were constructed to investigate the associations of patient demographics, plasma neurohormones, and echocardiographic variables with bradykinin genotype considered as a 3-level ordinate variable. Multiple variable linear regression models were also constructed; adjusting covariates included age, gender, body weight, height, and exercise history. Hardy-Weinberg equilibrium was tested by chi square analysis. Statistical significance was set at an alpha level of 0.05 for all analyses. Data are presented as mean ± standard deviation (SD) unless otherwise indicated.

## Results

### Participant characteristics

Participant clinical characteristics and medication use at the time of the study, according to genotype, are reported in Table 1. The percentage of women across groups was not different (homozygous +9 = 37%, heterozygous = 34.5%, homozygous −9 = 33.3%). There were no trends across groups for age, height, weight, or BMI. There also were no trends across groups for smoking or exercise history. There was a significant trend in the percentage of the groups using beta-blocker therapy with 88.9% in the homozygous +9, 79.0% in the heterozygous and 55.6% in the homozygous −9 groups. Further, there was a trend towards significant difference for the percentage of the groups using diuretic therapy with 75.6% in the homozygous +9, 63.2% in the heterozygous and 55.6% in the homozygous −9 groups.

### Plasma markers of neurohormonal activation and renin angiotensinaldosterone system (RAAS) activity

Table 2 shows the plasma neurohormone levels related to the RAAS activity. Because of the rightly skewed nature of these neurohormones, the log transformation of the absolute data was analyzed. Although there was not a statistically significant trend across the groups for angiotensin converting enzyme (ACE) inhibitor use, there was a trend towards reduced plasma levels of ACE for the homozygous −9 group as compared to the heterozygous or homozygous +9 groups (Table 2). There were no other significant differences across the groups for plasma levels of bradykinin, angiotensin II, atrial natriuretic peptide (ANP), brain natriuretic peptide (BNP), dopamine, epinephrine, or norepinephrine.

### Echocardiographic evaluation

The results of the functional and structural echocardiographic evaluation are shown in Table 3. There were no significant differences across the groups for EF, CO, or SV. There were also no significant trends across the groups for diastolic function measures including E and A wave velocities, E/A ratio, or deceleration time. There was a significant trend in PAPsys across the three genotype groups, being highest in the homozygous +9 group, intermediate in the heterozygous group, and lowest in the homozygous −9 group. The were no clinical or statistically significant trends across groups for LV mass or LV mass index, LV posterior wall thickness, LV dimension during systole and diastole or left atrial dimension.

**Figure 1:**
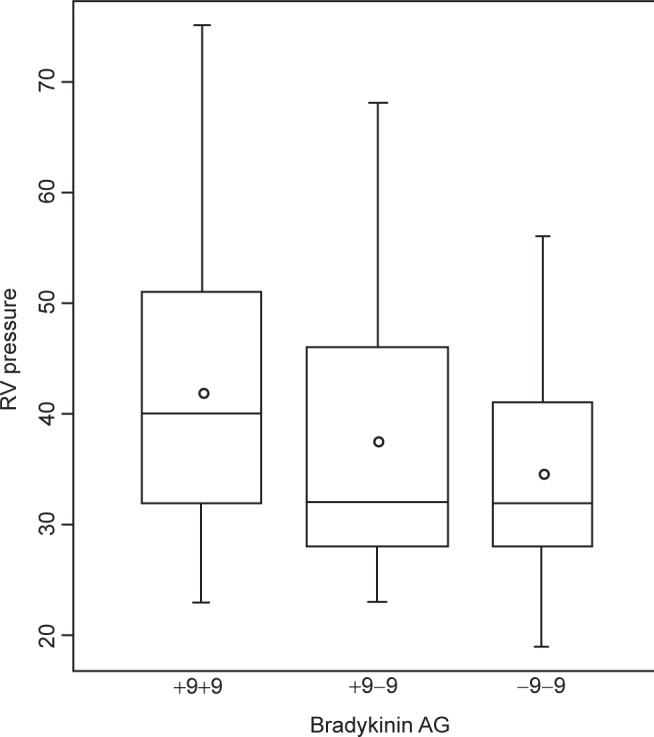
Relationship between bradykinin receptor B2 (BDKRB2) gene polymorphism and systolic pulmonary artery pressure (PAPsys) in heart failure (HF) patients. Patients who are homozygous +9 for the BDKRB2 demonstrate significantly higher PAPsys compared to those who are heterozygous or homozygous −9.

**Figure 2:**
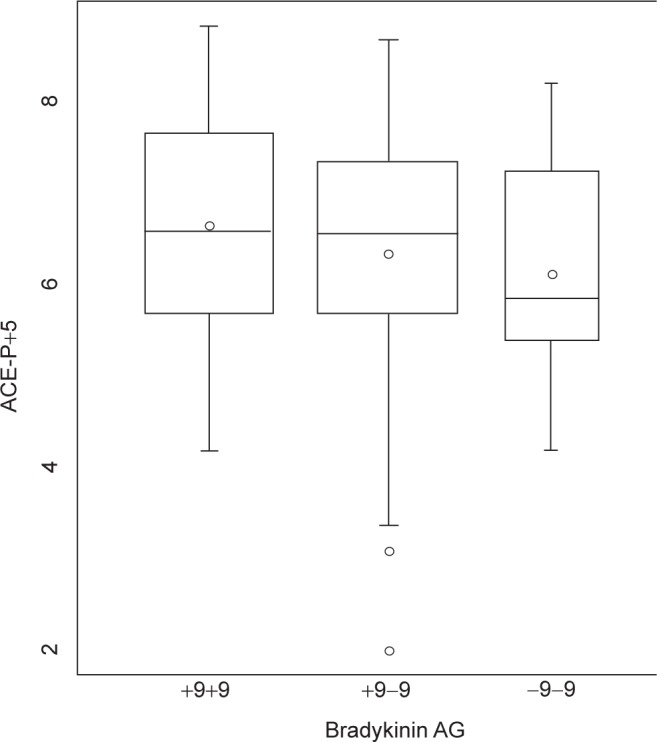
Relationship between bradykinin receptor B2 (BDKRB2) gene polymorphism and plasma angiotensin converting enzyme (ACE) level in heart failure (HF) patients. Although patients who are homozygous for the −9 BDKRB2 polymorphism demonstrate slightly lower plasma ACE levels, there was no significant trend across the groups.

To assess the independence of the genotypes on PAPsys, a multiple linear regression model was constructed, adjusting for age, gender, height, weight, and exercise history. After adjustment, there was a significant association between the level of PAPsys and the homozygous +9 genotype (p = 0.02) (Fig. 1). After adjusting for the same five covariates, an increased plasma ACE level demonstrated a trend toward significance with the homozygous +9 patients (Fig. 2). Further, after additionally adjusting for ACE inhibitor use, this association became significant (p = 0.04).

## Discussion

### Primary findings

This study evaluated the influence of the bradykinin B2 receptor genotype (BDKRB2) on pulmonary vascular pressure in patients with stable HF. Our results suggest patients homozygous for the +9 polymorphism demonstrate elevated PAPsys despite no significant differences in functional or structural cardiac parameters or heart failure severity. Further, the relationship between PAPsys and genotype polymorphism (p = 0.03) was strengthened after adjusting for potential confounding variables including age, height, weight, gender, and exercise history (p = 0.016). Although ACE levels were low due to chronic pharmacologic suppression, there remained evidence for a relationship between the BDKRB2 genotypes and plasma angiotensin converting enzyme levels. Further, the relationship between ACE levels and genotype was enhanced after adjusting for ACE inhibitor usage.

### Renin angiotensin-aldosterone system and regulation of vascular tone

Patients with primary systolic HF have a reduced cardiac reserve which is associated with neurohumoral activation of the renin-angiotensin-aldosterone system and peripheral vasoconstriction. Through blocking the enzyme responsible for conversion of angiotensin I to angiotensin II, ACE inhibitor therapy limits the generation of angiotensin II and thereby reduces the associated vasoconstriction and sodium and water retention. Therapy with ACE inhibition has known benefits in patients with heart failure including improvements in functional capacity, morbidity, and mortality.^25^–^28^ The administration of ACE inhibitor therapy in these patients causes systemic vasodilation, attributable to the loss of angiotensin II-mediated vasoconstriction.^29^ Recently, however, it has been suggested that the vasodilatory properties of bradykinin also contribute to the ACE inhibitor-induced vasodilation in both the radial and coronary arteries.^13^,^30^ This attenuation of vascular endothelial dysfunction by ACE inhibition appears to result from bradykinin potentiation.^13^

Bradykinin is a potent endothelium-dependent vasodilator that has a brief duration of action with a plasma half-life of approximately 15–30 seconds due to its rapid degradation by ACE.^9^,^31^ As such, ACE efficiently breaks down approximately 95% of bradykinin in a single passage through the pulmonary circulation.^32^ Exogenous administration of bradykinin induces vasodilation of epicardial coronary and resistance arteries in humans mediated in part by nitric oxide and endothelium-derived hyperpolarizing factor.^30^–^35^ The local and systemic vascular effects of exogenous bradykinin administration can be enhanced by ACE inhibition.^32^,^36^,^37^ In hypertensive patients and sodium-deplete volunteers, systemic infusion of a selective bradykinin B2 receptor antagonist attenuates the hypotensive effects of captopril similar to an angiotensin II type 1 receptor blocker.^38^ This suggests that some of the short-term hypotensive effects of ACE inhibition are mediated via augmentation of endogenous bradykinin through the BDKRB2.

### Bradykinin and vasculature function

Bradykinin demonstrates strong vasodilatory properties in the systemic circulation. Interestingly, little is known regarding the influence of bradykinin, bradykinin receptor regulation, or bradykinin receptor genotype variants on the regulation of pulmonary vascular tonality. In the systemic circulation, bradykinin plays an integral role in vascular regulation. Hornig and colleagues suggest that bradykinin contributes to flow mediated dilation of the brachial artery. These authors have shown that the independent infusion of the BDKRB2 antagonist icatibant results in a 33% reduction of flow-mediated dilation despite no significant effect on baseline arterial diameter or blood flow velocity. After infusion of the ACE inhibitor quinaprilat, flow-mediated dilation increased significantly by 46%. The coinfusion of icatibant and quinaprilat produced a reduction in flow-mediated dilation similar to icatibant alone suggesting bradykinin is fundamental in regulating vascular tone via the BDKRB2.^13^

Antagonism of the BDKRB2 also attenuates the short-term blood pressure lowering effects of ACE inhibition. Squire and colleagues have shown, in healthy men on a normo-sodium diet, that icatibant elicits a significant increase in mean arterial pressure. This would suggest a role for bradykinin in basal control of blood pressure. Further, when icatibant was co-administered with the ACE inhibitor perindoprilat the normal reduction in mean arterial pressure was attenuated despite no effect on neurohormonal responses to perindoprilat.^15^ Similarly, vasodilation in response to acetylcholine and bradykinin is blunted in resistance arteries of the forearm in patients with coronary artery disease with or without left ventricular dysfunction. Administration of quinaprilat enhanced the vasodilator response to bradykinin and suggests that ACE inhibition improves vasodilation through maximization of the bioavailability of bradykinin.^14^

In the pulmonary circulation, Taraseviciene-Stewart and colleagues examined the influence of a long-acting BDKRB2 agonist on pulmonary artery pressure in rats with severe pulmonary hypertension. These authors demonstrated that the administration of the stable BDKRB2 agonist B9972 reduced pulmonary artery pressure, right ventricular hypertrophy, and decreased number of apoptosis derived plexiform lesions in the obliterated vessels.^11^

Despite the understanding of bradykinins role in peripheral vascular tonality, little work has been done with regards to the specific polymorphisms of the BDKRB2 and vascular tonality either in the systemic or pulmonary circulations. In a prospective study, Dhamrait and colleagues demonstrated a significant contribution of the BDKRB2 +9/+9 polymorphism to the coronary artery disease risk associated with systemic hypertension.^39^ In a more recent study, Pretorius et al. suggest that participants with the +9/+9 BDKRB2 polymorphism have higher systolic blood pressure and vascular resistance which may contribute to the increased left ventricular mass associated with hypertension.^40^ These data suggest a role for the BDKRB2 +9/+9 polymorphism in changes in pulmonary pressures and the development of associated pulmonary hypertension. The results of the present study suggest that patients homozygous for the +9 polymorphism have higher PAPsys despite a higher proportion of these patients being treated with beta-blocker therapy. In this context two potential mechanisms for this difference are apparent. First, due to the genetic disposition of these patients, certain pharmacologic therapies may be less effective and require the addition of beta-blockers to stem the influence of co-morbidities. Pharmacogenetic interactions such as this have been suggested, however; the clarity of these interactions remains elusive and thus further research in this area is clearly warranted.^41^–^43^ Second, the patients homozygous for the −9 polymorphism demonstrated less beta-blocker therapy. This course of pharmacologic disease management in these patients may be the result of less symptomology or greater effectiveness of other pharmacologic strategies. As our understanding of pharmacogenomics widens, these interactions may become clearer.^41^–^43^

### Limitations

A potential limitation to this study is the use of echo-derived measures of pulmonary artery pressure. Although this methodology has been well validated by a number of investigators, it does not allow for the evaluation of pulmonary vascular resistance thus limiting the robustness of our analysis.^17^–^19^,^44^ As this study group consisted of a relatively homogeneous patient group, with subjects excluded with morbid obesity, extensive smoking history, heart rhythm abnormalities (e.g. afib), and pacer dependency, follow-up studies would be valuable on a more heterogenous HF cohort.

### Summary and conclusion

The results of the present study further the findings of others in a number of ways. First, despite maximization of pharmacologically mediated therapeutic regimens which include ACE inhibitor therapy, patients with heart failure continue to develop pulmonary arterial hypertension via a reactive component, most likely mediated through pulmonary arterial endothelial dysfunction. Second, independent of traditional measures cardiac structure and function or circulating plasma levels of neurohumoral markers indicative of activation of the renin-angiotensin-aldosterone system; patients who are homozygous +9 for the BDKRB2 polymorphism demonstrate significantly elevated levels of pulmonary artery systolic pressure. These results highlight the strength of the genetic contribution to the reactive component of secondary pulmonary hypertension in HF and provide more defined pathways to the development of pulmonary hypertension which may ultimately lead to more personalized treatment strategies in this population.

## Figures and Tables

**Table 1:** Participant Characteristics and Patient Medications According to Bradykinin B2 Receptor Gene Polymorphisms.

	HF (+9/+9) N = 46	HF (+9/−9) N = 58	HF (−9/−9) N = 27	Trend p-value
N (% Female)	17 (37.0%)	20 (34.5%)	9 (33.3%)	0.74
Age (yr)	54.0 ± 11.4	55.6 ± 12.2	58.7 ± 12.2	0.12
Height (cm)	171.8 ± 11.3	173.3 ± 9.0	172.6 ± 10.1	0.66
Weight (kg)	84.4 ± 18.6	85.4 ± 15.1	82.7 ± 20.0	0.76
BMI (kg/m^2^)	28.4 ± 4.9	28.4 ± 4.4	27.5 ± 4.8	0.47
Smoking Hx (pk/yrs)	2.5 ± 5.7	4.7 ± 9.7	3.8 ± 6.8	0.40
Exercise Hx (min/wk)	63.2 ± 85.2	86.8 ± 112.6	57.8 ± 72.1	0.98
NYHA Class	I = 26.1% II = 32.6% III = 34.8% IV = 6.5%	I = 37.9% II = 31.0% III = 22.4% IV = 8.6%	I = 40.7% II = 33.3% III = 18.5% IV = 7.4%	0.18
Medications				
ACE Inhibitors	34 (75.5%)	43 (74.1%)	19 (70.4%)	0.64
A-II Receptor Blockers	5 (11.1%)	7 (12.1%)	2 (7.4%)	0.68
β-Blockers	40 (88.9%)	45 (79.0%)	15 (55.6%)	0.002
Digitalis	33 (73.3%)	31 (54.4%)	19 (70.4%)	0.55
Diuretics	34 (75.6%)	36 (63.2%)	15 (55.6%)	0.07

**Abbrevations:** ACE, angiotensin converting enzyme; A-II, angiotensin II; BMI, body mass index; BSA, body surface area; Hx, history. Data are reported as Mean ± SD or count (percent).

**Table 2:** Differences in Plasma Neurohormonal Markers and Markers of RAAS Activity According to Bradykinin B2 Receptor Gene Polymorphisms.

	HF (+9/+9)	HF (+9/−9)	HF (−9/−9)	Trend p-value
Bradykinin-P (ng/mL)	21.3 ± 13.2	20.9 ± 17.7	23.1 ± 23.5	
ACE-P (U/L)	9.5 ± 10.7	7.1 ± 8.7	5.4 ± 6.4	
A-II (pg/mL)	13.0 ± 16.5	17.8 ± 24.3	9.2 ± 7.1	
ANP (pg/mL)	125 ± 119	162 ± 177	174 ± 158	
BNP (pg/mL)	346 ± 813	499 ± 1149	735 ± 1649	
Dopamine (pg/mL)	11.3 ± 5.9	13.0 ± 8.4	13.0 ± 10.2	
Epinephrine (pg/mL)	21.7 ± 19.9	23.6 ± 18.0	16.7 ± 11.8	
Norepinephrine (pg/mL)	346 ± 248	401 ± 358	389 ± 287	
Log: Bradykinin-P	2.90 ± 0.58	2.81 ± 0.66	2.85 ± 0.74	0.68
Log: ACE-P + 5	2.46 ± 0.62	2.30 ± 0.62	2.20 ± 0.52	0.06
Log: A-II	2.09 ± 0.91	2.27 ± 1.06	1.92 ± 0.89	0.67
Log: ANP + 10	4.57 ± 0.84	4.68 ± 0.99	4.86 ± 0.88	0.21
Log: BNP + 1	4.79 ± 1.50	4.72 ± 1.82	5.24 ± 1.70	0.36
Log: Dopamine	2.33 ± 0.43	2.43 ± 0.49	2.41 ± 0.49	0.41
Log: Epinephrine	2.78 ± 0.74	2.90 ± 0.73	2.61 ± 0.62	0.52
Log: Norepinephrine	5.46 ± 1.07	5.56 ± 1.05	5.66 ± 0.90	0.43

**Abbrevations:** ACE, angiotensin converting enzyme; AII, angiotensin II; ANP, atrial naturietic peptide; BNP, brain naturietic peptide. Data are reported as Mean ± SD and logarithmic transformation of the Mean ± SD.

**Table 3:** Differences in Functional and Structural Echocardiographic Measures According to Bradykinin B2 Receptor Gene Polymorphisms.

	HF (+9/+9)	HF (+9/−9)	HF (−9/−9)	Trend p-value
Functional Measures				
LV Ejection Fraction (%)	27.3 ± 9.2	29.8 ± 11.8	30.5 ± 14.1	0.21
CO (L/min)	5.25 ± 1.46	4.88 ± 1.29	5.17 ± 1.40	0.67
Cardiac Index (L/m/m^2^)	2.67 ± 0.61	2.47 ± 0.71	2.74 ± 0.64	0.92
SV (cc)	77.1 ± 26.1	73.1 ± 24.6	82.5 ± 23.3	0.55
E (m/sec)	0.83 ± 0.28	0.75 ± 0.29	0.72 ± 0.24	0.11
A (m/sec)	0.72 ± 0.27	0.64 ± 0.25	0.80 ± 0.33	0.48
E/A	1.47 ± 1.21	1.38 ± 0.91	1.05 ± 0.65	0.15
Deceleration Time (msec)	190.6 ± 60.1	195.6 ± 62.4	215.9 ± 81.8	0.18
RV Systolic Pressure (mmHg)	41.9 ± 13.0	37.6 ± 12.0	34.6 ± 10.9	0.03
Structural Measures				
LV Mass (g)	276.9 ± 77.8	280.1 ± 95.9	281.3 ± 94.3	0.84
LV Mass Index (g/m^2^)	139.8 ± 38.3	138.8 ± 48.9	152.1 ± 52.8	0.38
LV Posterior Wall (mm)	9.57 ± 1.48	10.0 ± 1.80	9.73 ± 1.64	0.56
LV Dimension, Dia (mm)	67.0 ± 10.5	64.4 ± 9.6	67.0 ± 10.7	0.80
LV Dimension, Sys (mm)	57.4 ± 12.9	54.9 ± 13.3	54.0 ± 12.3	0.31
LA Dimension (mm)	46.6 ± 7.63	48.7 ± 8.45	46.7 ± 11.0	0.84

**Abbrevations:** LV, left ventricle; CO, cardiac output; SV, stroke volume; E, peak early mitral inflow velocity; A, peak late mitral inflow velocity; Dia, diastole; Sys, systole; LA, left atria. Data are reported as Mean ± SD.

## References

[1] Enriquez-Sarano M, Maurice Rossi M, Andrea Seward M, James B, Bailey P, Kent R (1997). Determinants of Pulmonary Hypertension in Left Ventricular Dysfunction. Journal of the American College of Cardiology.

[2] Tribouilloy C, Enriquez-Sarano M, Rossi A, Tajik A, Seward J (1997). Determinants of the pulmonary artery pressure rise in left ventricular dysfunction. Cardiologia.

[3] Cheever K (2005). An overview of pulmonary arterial hypertension: risks, pathogenesis, clinical manifestations, and management. J Cardiovasc Nurs.

[4] Lefebvre F, Prefontaine A, Calderone A, Caron A (2006). Modification of the pulmonary renin-angiotensin system and lung structural remodeling in congestive heart failure. Clin Sci.

[5] Stewart JM, Gera L, Chan DC, Whalley ET, Hanson WL, Zuzack JS (1997). Potent, long-acting bradykinin antagonists for a wide range of applications. Can J Physiol Pharmacol.

[6] Drummond GR, Cocks TM (1995). Endothelium-dependent relaxation to the B1 kinin receptor agonist des-Arg9-bradykinin in human coronary arteries. Br J Pharmacol.

[7] McLean PG, Perretti M, Ahluwalia A (2000). Kinin B1 receptors and the cardiovascular system: regulation of expression and function. Cardiovasc Res.

[8] Marin-Castano ME, Schanstra JP, Neau E, Praddaude F (2002). Induction of functional bradykinin B1-receptors in normotensive rats and mice under chronic angiotensin-converting enzyme inhibitor treatment. Circulation.

[9] Cockcroft JR, Chowienczyk PJ, Brett SE, Bender N, Ritter JM (1994). Inhibition of bradykinin-induced vasodilation in human forearm vasculature by icatibant, a potent B2-receptor antagonist. Br J Clin Pharmacol.

[10] Brown NJ, Gainer JV, Murphey LJ, Vaughan DE (2000). Bradykinin stimulates tissue plasminogen activator release from human forearm vasculature through B2 receptor-dependent, NO synthase-independent, and cyclooxygenase-independent pathway. Circulation.

[11] Taraseviciene-Stewart L, Scerbavicius R, Stewart JM (2005). Treatment of severe pulmonary hypertension: A bradykinin receptor 2 agonist B9972 causes reduction of pulmonary artery pressure and right ventricular hypertrophy. Peptides.

[12] Kammerer S, Braun A, Arnold N, Roscher AA (1995). The human bradykinin B2 receptor gene: full length cDNA, genomic organization and identification of the regulatory region. Biochem Biophys Res Commun.

[13] Hornig B, Kohler C, Drexler H (1997). Role of bradykinin in mediating vascular effects of angiotensin converting exzyme inhibitors in humans. Circulation.

[14] Benacerraf S, Carville C, Adnot S (1999). Improvement of bradykinin endothelium-mediated vasodilation of forearm resistance circulation by Quinaprilat in patients with coronary artery disease with or without left ventricular dysfunction. J Cardiovasc Pharmacol.

[15] Squire IB, O’Kane KP, Anderson N, Reid JL (2000). Bradykinin B2 receptor antagonism attenuates blood pressure response to acute angiotensinconverting enzyme inhibition in normal men. Hypertension.

[16] Schiller NB, Shah PM, Crawford M (1989). Recommendations for quantification of the left ventricle by two-dimensional echocardiography. American Society of Echocardiography committee on standards, subcommittee on quantitation of two-dimensional echocardiograms. J Am Soc Echocardiogr.

[17] Yock PG, Popp RL (1984). Noninvasive estimation of right ventricular systolic pressure by Doppler ultrasound in patients with tricuspid regurgitation. Circulation.

[18] Berger M, Haimowitz A, van Tosh A, Berdoff RL, Goldberg E (1985). Quantitative assessment of pulmonary hypertension in patients with tricuspid regurgitation using continuous wave Doppler ultrasound. J Am Coll Cardiol.

[19] Currie PJ, Sweard JB, Chan KL, Fyfe DA, Hagler DJ, Mair DD (1985). Continuous wave Doppler determination of right ventricular pressure: A simultaneous Doppler-catheterzation study in 127 patients. J Am Coll Cardiol.

[20] Lanzarini L, Fontana A, Lucca E, Campana C, Klersy C (2002b). Noninvasive estimation of both systolic and diastolic pulmonary artery pressure from Doppler analysis of tricuspid regurgitant velocity spectrum in patients with chronic heart failure. Am Heart J.

[21] Troy BL, Pombo J, Rackley CE (1972). Measurement of left ventricular wall thickness and mass by echocardiography. Circulation.

[22] Lewis JF, Kuo LC, Nelson JG, Limacher MC, Quinones MA (1984). Pulsed Doppler echocardiographic determination of stroke volume and cardiac output: clinical validation of two new methods using the apical window. Circulation.

[23] Parisi AF, Moynihan PF, Feldman CL, Folland ED (1979). Approaches to determination of left ventricular volume and ejection fraction by real-time two-dimensional echocardiography. Clin Cardiol.

[24] Braun A, Kammerer S, Bohme E, Muller B, Roscher AA (1995). Identification of polymorphic sites of the human bradykinin B2 receptor gene. Biochem Biophys Res Commun.

[25] Group CS (1987). Effects of enalapril on mortalit in severe congestive heart failure. Results of the Cooperative North Scandinavian Enalapril Survival Study (CONSENSUS). N Engl J Med.

[26] Cohn JN, Johnson G, Zeische S (1991). A comparison of enalapril with hydralazine-isosorbide dinitrate in the treatment of chronic congestive heart failure. N Engl J Med.

[27] Investigators AS (1993). Effect of ramipril on mortality and morbidity of survivors of acute myocardial infarction with clinical evidence of heart failure. Lancet.

[28] Abraham MR, Olson LJ, Joyner MJ, Turner ST, Beck KC, Johnson BD (2002). Angiotensin-converting enzyme genotype modulates pulmonary function and exercise capacity in treated patients with congestive stable heart failure. Circulation.

[29] Flapan AD, Shaw TR, Edwards CR, Davies E, Williams BC (1992). Contrasting patterns of arterial and venous dilation after intravenous captopril in patients with chronic cardiac failure and their relationship to plasma angiotensin II concentrations. Am Heart J.

[30] Kuga T, Mohri M, Egashira K (1997). Bradykinin-induced vasodilation of human coronary arteries in vivo: role of nitric oxide and angiotensinconverting enzyme. J Am Coll Cardiol.

[31] Cherry PD, Furchgott R, Zawadzki J, Jothianandan D (1982). Role of endothelial cells in relaxation of isolated arteries by bradykinin. Proc Natl Acad Sci USA.

[32] Bonner G, Preis S, Schunk U, Wagmann M, Chrosch R, Toussaint C (1992). Effect of bradykinin on arteries and veins in systemic and pulmonary circulation. J Cardiovasc Pharmacol.

[33] O’Kane KP, Webb DJ, Collier JG, Vallance PJ (1994). Local L-NG-monomethyl-agrinine attenuates the vasodilator action of bradykinin in the human forearm. Br J Clin Pharmacol.

[34] Groves P, Kurz S, Just H, Drexler H (1995). Role of endogenous bradykinin in human coronary vasomotor control. Circulation.

[35] Honing ML, Smits P, Morrison PJ, Rakelinik TJ (2000). Bradykinin-induced vasodilation of human forearm resistance cessels is primarily mediated by endothlium-dependent hyperpolarization. Hypertension.

[36] Benjamin N, Cockcroft JR, Collier JG, Dollery CT, Ritter JM, Webb DJ (1989). Local inhibition of converting enzyme and vascular responses to angiotensin and bradykinin in the human forearm. J Physiol.

[37] Bonner G, Preis S, Schunk U, Toussaint C, Kauffmann W (1990). Hemodynamic effects of bradykinin and pulmonary circulation in healthy and hypertensive humans. J Cardiovasc Pharmacol.

[38] Gainer JV, Morrow JD, Loveland A, King DJ, Brown NJ (1998). Effect of bradykinin-receptor blockade on the response to angiotensinconverting-enzyme inhibitor in normotensive and hypertensive subjects. N Engl J Med.

[39] Dhamrait SS, Payne JR, Li P, Humphries SE, Montgomery HE (2003). Variation in bradykinin receptor genes increases the cardiovascular risk associated with hypertension. Eur Heart J.

[40] Pretorius MM, Gainer JV, Van Guilder GP (2007). The bradykinin type 2 receptor BE1 polymorphism and ethnicity influences systolic blood pressure and vascular resistance. Clin Pharmacol Ther.

[41] McNamara DM, Holubkov R, Janosko K (2001). Pharmacogenetic interactions between beta-blocker therapy and the angiotensin-converting enzyme deletion polymorphism in patients with congestive heart failure. Circulation.

[42] Roden DM, Brown NJ (2001). Prescribing Genotyping: Not yet ready for prime time, but getting there. Circulation.

[43] McNamara DM, MacGowan GA, London B (2002). Clinical importance of beta-adrenoceptor polymorphisms in cardiovascular disease. Am J Pharmacogenomics.

[44] Lanzarini L, Fontana A, Lucca E, Campana C, Klersy C (2002a). Noninvasive estimation of both systolic and diastolic pulmonary artery pressure from Doppler analysis of tricuspid regurgitant velocity spectrum in patients with chronic heart failure. Am Heart J.

